# Two New Highly Oxygenated Spirostanol Saponins from *Paris polyphylla* var. *stenophylla*

**DOI:** 10.1007/s13659-016-0102-x

**Published:** 2016-06-02

**Authors:** Ling-Yu Jin, Ting-Xiang Lu, Xu-Jie Qin, Wei Ni, Huan Yan, Yu Chen, Hui Liu, Hong-Ping He, Hai-Yang Liu

**Affiliations:** College of Pharmacy and Chemistry, Dali University, Dali, 671000 China; State Key Laboratory of Phytochemistry and Plant Resources in West China, Kunming Institute of Botany, Chinese Academy of Sciences, Kunming, 650201 China; University of Chinese Academy of Sciences, Beijing, 100049 China

**Keywords:** *Paris polyphylla* var. *stenophylla*, Liliaceae, Spirostanol saponins, Paristenoside A, Paristenoside B

## Abstract

**Abstract:**

Phytochemical investigation of the rhizomes of *Paris polyphylla* var. *stenophylla* led to the isolation of two new highly oxygenated spirostanol saponins, named paristenosides A (**1**) and B (**2**), together with seven known compounds. Their structures were established mainly on the base of NMR spectroscopic techniques and mass spectrometry, as well as chemical methods. In addition, the cytotoxicity of the two new saponins was tested.

**Graphical Abstract:**

Two new highly oxygenated spirostanol saponins, paristenosides A (**1**) and B (**2**), were isolated from the rhizomes of *Paris polyphylla* var. *stenophylla*. Their structures were established mainly based on NMR spectroscopic techniques and mass spectrometry, as well as chemical methods.
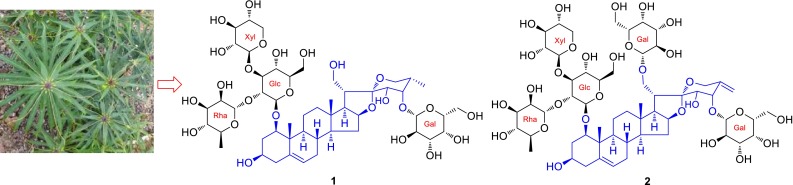

**Electronic supplementary material:**

The online version of this article (doi:10.1007/s13659-016-0102-x) contains supplementary material, which is available to authorized users.

## Introduction

The genus *Paris* (Liliaceae) comprises about 24 species, which grows as perennial rhizomatous herbs in the Eurasian continent from temperate zone to the tropics [1]. Rhizoma Paridis (‘Chonglou’ in Chinese), the dried rhizomes of *P. polyphylla* var. *yunnanensis* and *P. polyphylla* var. *chinensis*, have been recorded in the Pharmacopoeia of the People’s Republic of China as a traditional Chinese medicine for the treatment of traumatic injuries, insect and snake bites, and sore throat [2]. Plants of the genus *Paris* are known as a rich source of steroidal saponins, which have attracted great interests for their structural diversity and various bioactivities such as anti-tumor [3, 4], hemostatic [5], and antifungal [6] effects. In recent years, our group focuses the research on the chemical constituents, bioactivity, and sustainable utilization of resources of the genus [7–11].

**Fig. 1:**
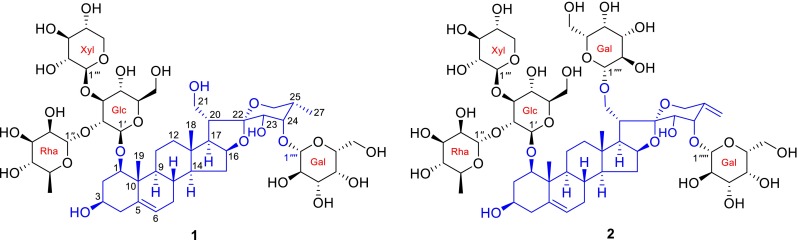
Chemical structures of paristenosides A (**1**) and B (**2**)

*P. polyphylla* var. *stenophylla* Franch. is one of the variations of *P. polyphylla* and has been used as a herbal medicine to treat skin furuncle and skin tinea, stop bleeding, and move clean blood and bad blood out by the Yi nationality of Liangshan in Sichuan Province [12]. Due to having certain resources, its rhizomes have been as the main steam commodities of Rhizoma Paridis. However, there are a few reports about the research of its material basis [13]. In order to clarify its chemical quality, we performed a phytochemical investigation of the rhizomes of *P. polyphylla* var. *stenophylla*. As a result, two new polyhydroxylated steroidal saponis, named paristenosides A (**1**) and B (**2**), were isolated from the title species. Meanwhile, seven known compounds were obtained and identified as paris saponin H (**3**) [14], Tg (**4**) [15], Pb (**5**) [16], Th (**6**) [17], Methyl-Th (**7**) [18], parispseudoside A (**8**) [19] and *β*-ecdysone (**9**) [16]. In the current paper, we report the isolation, structural elucidation, and cytotoxicity of the two new compounds (Fig. 1).

## Results and Discussion

**Fig. 2:**
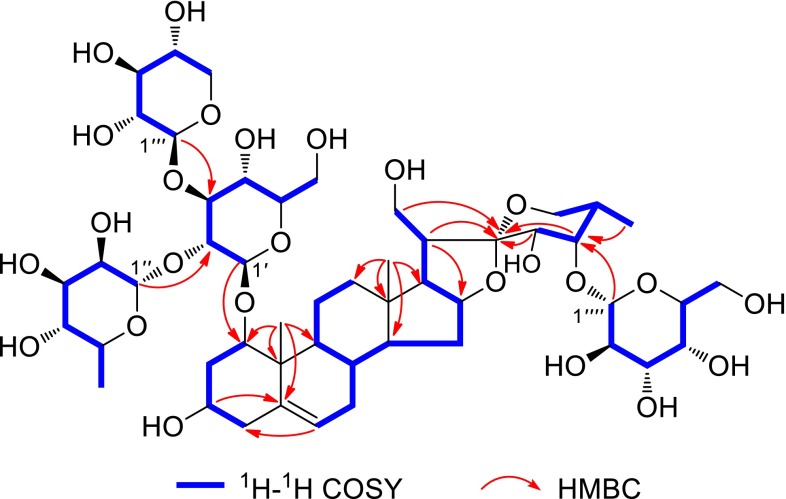
^1^H-^1^H COSY and Key HMBC correlations of paristenoside A (**1**)

**Fig. 3:**
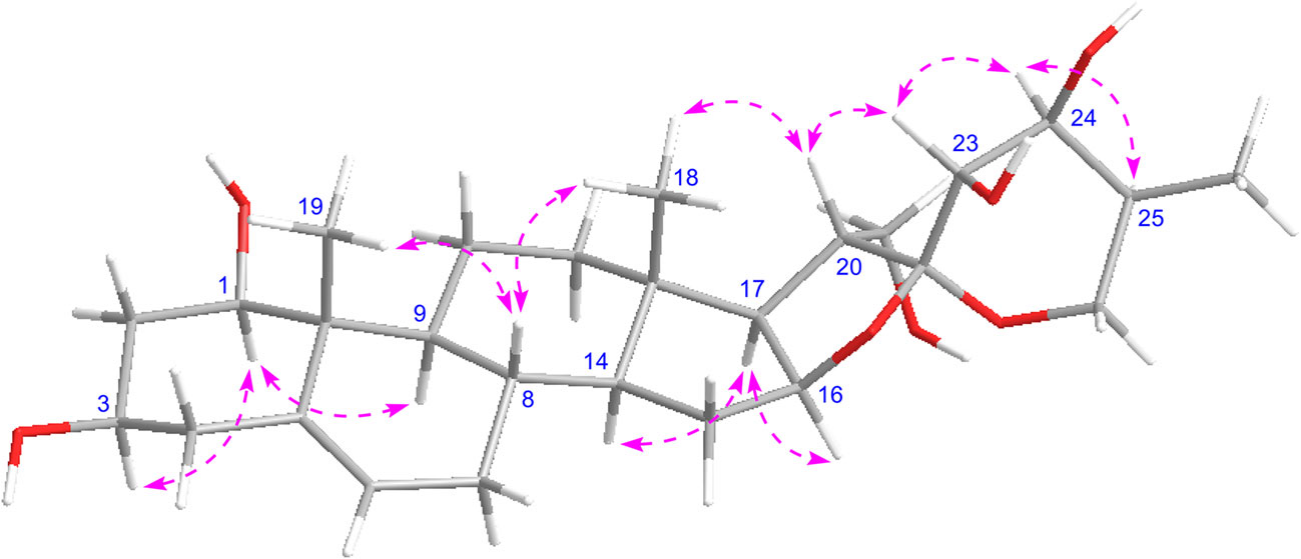
Key ROESY correlations for the aglycone moiety of paristenoside A (**1**)

Paristenoside A (**1**) was isolated as a white amorphous powder. Its molecular formula, C_50_H_80_O_25_, was assigned by the positive-ion HR-ESI-MS (*m*/*z* 1103.4881 [M + Na]^+^, calcd for 1103.4881) and ^13^C NMR data (Tables 1, 2). The IR spectrum displayed absorption bands ascribable to hydroxy (3426 cm^−1^) and olefinic bond (1631 cm^−1^) functionalities. The ^13^C NMR spectrum showed signals for a ketal carbon at *δ*_C_ 111.5, a trisubstituted olefin carbons at *δ*_C_ 139.4 (s) and 124.6 (d), and three methyls at *δ*_C_ 17.0, 15.0, and 13.1, which were characteristic of a ∆^5,6^-spirostanol skeleton as an aglycone [7]. Its ^1^H NMR spectrum showed two singlet signals for two tertiary methyls at *δ*_H_ 1.38 and 1.13, one doublet signal for a secondary methyl at *δ*_H_ 1.03 (d, *J* = 6.8 Hz), an olefinic proton at *δ*_H_ 5.55 (br d, *J* = 5.5 Hz), as well as four anomeric protons at *δ*_H_ 6.41 (br s), 5.24 (d, *J* = 7.8 Hz), 4.91 (d, *J* = 7.6 Hz), and 4.78 (d, *J* = 7.7 Hz), which suggested the presence of four sugar units. In addition, one methyl at *δ*_H_ 1.71 (d, *J* = 6.0 Hz) was the methyl of one 6-deoxyhexopyranose residue. Comparison of the ^1^H and ^13^C NMR spectra of **1** with those of padelaoside A [20] revealed that **1** possessed the same polyhydroxylated aglycone [(23*S*,24*S*,25*S*)-spirost-5-ene-1*β*,3*β*,21,23,24-pentol] as that of padelaoside A, which could be further verified by detailed 2D NMR data analysis (Figs. 2, 3). Furthermore, the result of acid hydrolysis and GC analysis of **1** with its corresponding trimethylsilated l-cysteine adducts afforded d-glucose, d-galactose, d-xylose, and l-rhamnose. The large coupling constants (^3^*J*_1,2_ > 7 Hz) were consistent with *β*-configuration for the glucose, galactose, and xylose, while the *α*-configuration for the rhamnose was deduced by comparing its ^13^C NMR spectroscopic data for C-3″ (*δ*_C_ 72.4) and C-5″ (*δ*_C_ 69.5) with those reported in the literature [21]. The sequence and binding sites of the sugar units were determined by following HMBC correlations: *δ*_H_ 6.41 (H-1″ of Rha) with *δ*_C_ 76.3 (C-2′ of Glc), *δ*_H_ 4.91 (H-1″′ of Xyl) with *δ*_C_ 88.3 (C-3′ of Glc), *δ*_H_ 4.78 (H-1′ of Glc) with *δ*_C_ 83.9 (C-1 of the aglycone), and *δ*_H_ 5.24 (H-1″″ of Gal) with *δ*_C_ 81.8 (C-24 of the aglycone). Assignment of all proton and carbon resonances was achieved by interpretation of ^1^H-^1^H COSY, HSQC, and HMBC spectra. Based on the above information, the structure of paristenoside A (**1**) was elucidated as 24-*O*-*β*-d-galactopyranosyl-(23*S*,24*S*,25*S*)-spirost-5-ene-1*β*,3*β*,21,23,24-pentol-1-*O*-*α*-l-rhamnopyranosyl-(1 → 2)-[*β*-d-xylopyranosyl-(1 → 3)]-*β*-d-glucopyranoside.

**Table 1:** ^1^H and ^13^C NMR data for the aglycone portions of paristenosides A **(1**) and B (**2**) (in C_5_D_5_N, 600 MHz)

No.	Paristenoside A		Paristenoside B	
	*δ*_C_	*δ*_H_ (mult., *J* in Hz)^a^	*δ*_C_	*δ*_H_ (mult., *J* in Hz)^a^
1	83.9 (CH)	3.85 (dd, 12.0, 3.8)	84.6 (CH)	3.77 (dd, 11.9, 3.9)
2a	37.7 (CH_2_)	2.65	38.0 (CH_2_)	2.63
2b		2.38 (q, 11.9)		2.39 (q, 12.0)
3	67.9 (CH)	3.74 (m)	67.9 (CH)	3.73 (m)
4a	43.6 (CH_2_)	2.66 (br t, 12.2)	43.7 (CH_2_)	2.65 (t, 12.6)
4b		2.54 (dd, 11.6, 3.7)		2.52 (dd, 12.0, 3.5)
5	139.4 (C)		139.3 (C)	
6	124.6 (CH)	5.55 (br d, 5.5)	124.7 (CH)	5.52 (br d, 5.3)
7a	31.7 (CH_2_)	1.67 (m)	31.7 (CH_2_)	1.72 (m)
7b		1.40 (m)		1.43 (m)
8	33.0 (CH)	1.48 (m)	32.9 (CH)	1.45 (m)
9	50.0 (CH)	1.61 (m)	50.2 (CH)	1.59 (m)
10	42.6 (C)		42.6 (C)	
11a	24.0 (CH_2_)	2.82 (m)	23.9 (CH_2_)	2.77 (m)
11b		1.61 (m)		1.47 (m)
12a	40.2 (CH_2_)	1.92 (m)	39.9 (CH_2_)	1.88 (br d, 9.5)
12b		1.48		1.46
13	41.0 (C)		40.9 (C)	
14	56.9 (CH)	1.15 (m)	56.9 (CH)	1.14 (m)
15a	32.4 (CH_2_)	1.80 (m)	32.2 (CH_2_)	1.80 (m)
15b		1.47		1.43
16	83.1 (CH)	4.58	83.3 (CH)	4.53
17	58.1 (CH)	2.00 (t, 7.2)	58.1 (CH)	1.94 (t, 7.5)
18	17.0 (CH_3_)	1.13 (s)	16.8 (CH_3_)	1.03 (s)
19	15.0 (CH_3_)	1.38 (s)	14.9 (CH_3_)	1.32 (s)
20	46.0 (CH)	3.34 (br t, 5.6)	43.5 (CH)	3.42 (q, 6.7)
21a	62.5 (CH_2_)	4.20 (m)	69.8 (CH_2_)	4.43 (m)
21b		4.04 (m)		3.94 (m)
22	111.5 (C)		111.3 (C)	
23	71.1 (CH)	4.10 (m)	71.1 (CH)	4.34 (d, 2.6)
24	81.8 (CH)	4.17 (m)	82.0 (CH)	4.68 (d, 2.6)
25	35.1 (CH)	1.93 (m)	143.4 (C)	
26a	61.4 (CH_2_)	3.98 (m)	61.4 (CH_2_)	4.84 (d, 12.2)
26b		3.35 (m)		3.98 (d, 12.2)
27a	13.1 (CH_3_)	1.03 (d, 6.8)	113.8 (CH_2_)	5.04 (s)
27b				4.96 (s)

^a^Overlapped signals are reported without designating multiplicity

**Table 2:** ^1^H and ^13^C NMR data for the sugar moieties of paristenosides A **(1**) and B (**2**) (in C_5_D_5_N, 600 MHz)

No.	Paristenoside A		No.	Paristenoside B	
	*δ*_C_	*δ*_H_ (mult., *J* in Hz)^a^		*δ*_C_	*δ*_H_ (mult., *J* in Hz)^a^
1-Glc-1′	99.7 (CH)	4.78 (d, 7.7)	1-Glc-1′	100.2 (CH)	4.73 (d, 7.6)
2′	76.3 (CH)	4.13 (m)	2′	76.2 (CH)	4.06
3′	88.3 (CH)	4.03 (m)	3′	88.2 (CH)	4.01 (t, 9.2)
4′	70.1 (CH)	3.73 (m)	4′	69.8 (CH)	3.83 (t, 9.2)
5′	77.7 (CH)	3.77 (m)	5′	77.6 (CH)	3.74 (m)
6′a	63.1 (CH_2_)	4.45 (m)	6′a	63.1 (CH_2_)	4.45 (m)
6′b		4.14 (m)	6′b		4.22 (m)
2′-Rha-1″	101.6 (CH)	6.41 (br s)	2′-Rha-1″	101.6 (CH)	6.34 (br s)
2″	72.4 (CH)	4.75 (m)	2″	72.4 (CH)	4.75
3″	72.4 (CH)	4.57 (m)	3″	72.3 (CH)	4.55 (dd, 9.5, 3.3)
4″	74.1 (CH)	4.29 (m)	4″	74.0 (CH)	4.28 (t, 9.4)
5″	69.5 (CH)	4.81 (dq, 9.2, 6.3)	5″	69.4 (CH)	4.78 (dq, 9.2, 6.2)
6″	19.2 (CH_3_)	1.71 (d, 6.0)	6″	19.1 (CH_3_)	1.70 (d, 6.1)
3′-Xyl-1″′	105.1 (CH)	4.91 (d, 7.6)	3′-Xyl-1″′	105.1 (CH)	4.90 (d, 7.7)
2″′	74.6 (CH)	3.95 (t, 8.1)	2″′	74.6 (CH)	3.92 (t, 8.2)
3″′	78.3 (CH)	4.06 (t, 8.9)	3″′	78.3 (CH)	4.05
4″′	70.5 (CH)	4.10 (m)	4″′	70.5 (CH)	4.08 (m)
5″′a	67.1 (CH_2_)	4.23 (dd, 11.3, 5.0)	5″′a	67.1 (CH_2_)	4.23 (m)
5″′b		3.66 (t, 10.6)	5″′b		3.66 (t, 10.8)
24-Gal-1″″	106.3 (CH)	5.24 (d, 7.8)	21-Gal-1″″	105.1 (CH)	4.85 (d, 7.5)
2″″	72.4 (CH)	4.56	2″″	72.3 (CH)	4.44
3″″	75.3 (CH)	4.14 (m)	3″″	75.3 (CH)	4.12 (dd, 9.4, 3.2)
4″″	70.0 (CH)	4.53 (m)	4″″	70.1 (CH)	4.52
5″″	76.9 (CH)	4.06 (br t, 8.8)	5″″	76.9 (CH)	4.06
6″″a	62.0 (CH_2_)	4.47 (m)	6″″	62.2 (CH_2_)	4.38 (2H, br d, 5.9)
6″″b		4.37 (m)	24-Gal-1″″′	103.9 (CH)	5.82 (d, 8.2)
			2″″′	70.7 (CH)	4.64 (dd, 8.1, 2.9)
			3″″′	75.2 (CH)	4.68 (m)
			4″″′	71.1 (CH)	4.59 (br d, 3.2)
			5″″′	76.9 (CH)	4.06
			6″″′	62.3 (CH_2_)	4.31 (2H, dd, 11.0, 5.7)

^a^Overlapped signals are reported without designating multiplicity

Paristenoside B (**2**) was obtained as a white amorphous powder with a molecular formula of C_56_H_88_O_30_, which was deduced from the positive HR-ESI-MS (*m*/*z* 1258.5689 [M + NH_4_]^+^, calcd for 1258.5699) and ^13^C NMR data (Tables 1, 2). The ^1^H and ^13^C NMR data suggested that compound **2** was also a highly oxygenated spirostanol glycoside. A comparison of the NMR spectra of **2** with those of parisyunnanoside I [22] disclosed that they both had the same aglycone and sugar chain linkages at C-1 and C-21, except for the replacement of the fucose by a galactose at C-24 in the latter. This was further supported by the HMBC correlation between *δ*_H_ 5.82 (H-1″″′ of Gal) with *δ*_C_ 82.0 (C-24 of the aglycone). The other parts of **2** were identical to those of parisyunnanoside I as confirmed by detailed analysis of its 2D NMR experiments. Therefore, the structure of paristenoside B (**2**) was established as 21-*O*-*β*-d-galactopyranosyl-24-*O*-*β*-d-galactopyranosyl-(23*S*,24*S*)-spirost-5,25(27)-diene-1*β*,3*β*,21,23,24-pentol-1-*O*-*α*-l-rhamnopyranosyl-(1 → 2)-[*β*-d-xylopyranosyl-(1 → 3)]-*β*-d-glucopyranoside.

The phytochemical investigation of the rhizomes of *P. polyphylla* var. *stenophylla* led to the identification of eight steroidal saponins and one ecdysone, including two new highly oxygenated spirostanol glycosides. Compounds **1** and **2** are a further addition to the diverse and complex steroidal saponins. Our results and the literature [13] suggested that *P. polyphylla* var. *stenophylla* contained the same bioactive components as those in the legally original plants of Rhizoma Paridis. However, whether the rhizomes of *P. polyphylla* var. *stenophylla* can be used as the original plant of Rhizoma Paridis are still valuable for us to further research. Considering cytotoxicity of steroidal glycosides, paristenosides A (**1**) and B (**2**) were evaluated for their cytotoxicity against two human cancer cell lines (HEK293 and HepG2) by using MTT method. Unfortunately, none of them showed significant activity at the concentration of 20 μM.

## Experimental Section

### General Experimental Procedures

Optical rotations were recorded on a JASCO P-1020 digital polarimeter. IR spectra were obtained on Bruker Tensor-27 infrared spectrophotometer with KBr pellets. ESI-MS spectra were recorded on a Bruker HCT/Esquire spectrometer. HR-ESI-MS were obtained on an Agilent 6200 Q-TOF MS system. 1D and 2D NMR spectra were obtained on a Bruker Avance III 600 MHz spectrometer in C_5_D_5_N; chemical shifts are given in *δ* (ppm), and coupling constants are reported in Hz. Column chromatography (CC) was performed on silica gel (200–300 mesh, Qingdao Puke Chemical Co. Ltd., China) and Rp-18 gel (40–63 μm, Merck, Darmstadt, Germany). GC analysis was performed on a HP5890 gas chromatograph equipped with an H_2_ flame ionization detector. Semi-preparative HPLC was run on Agilent 1100 liquid chromatograph equipped with a Zorbax SB-C18 column (5 μm, 25 cm × 9.4 mm) and a diode array detector (DAD). TLC was performed on HSGF_254_ (0.2 mm, Qingdao Puke Chemical Co. Ltd., China). Fractions were monitored by TLC and spots were visualized by heating silica gel plates sprayed with 10 % H_2_SO_4_ in EtOH.

### Plant Material

The rhizomes of *P. polyphylla* var. *stenophylla* were collected in Octobor 2014 from Zhaoyang District, Zhaotong, Yunnan province, China. The plant material was authenticated by Dr. Yun-Heng Ji, Kunming Institute of Botany, Chinese Academy of Sciences. A voucher specimen (No. HY0023) was deposited at the State Key Laboratory of Phytochemistry and Plant Resources in West China, Kunming Institute of Botany, Chinese Academy of Sciences.

### Extraction and Isolation

The air-dried and powdered rhizomes of *P. polyphylla* var. *stenophylla* (370 g) were extracted three times with 75 % EtOH under reflux and then the solvent was evaporated under reduced pressure to afford a crude extract (10 g). The crude extract was subjected to a silica gel column and eluted with a CHCl_3_–MeOH (15:1 → 1:2, v/v) gradient solvent system to yield seven fractions (A–G). Fr. C (1.1 g) was chromatographed through a silica gel column (CHCl_3_–MeOH, 15:1 → 1:1) and purified by semi-prep. HPLC (CH_3_CN-H_2_O, 40:60, v/v) to yield **3** (35 mg). Fr. E (1.5 g) was separated by an Rp-18 column (CH_3_OH–H_2_O, 50:50 → 70:30, v/v) to get **4** (9 mg) and **5** (30 mg). Fr. F (1.5 g) was separated by a silica gel column, eluting with CHCl_3_–MeOH (10:1 → 1:1) and was further purified by semi-prep. HPLC with the mobile phase of CH_3_CN–H_2_O (30:70, v/v) to obtain **1** (9 mg), **2** (5 mg), **6** (43 mg), **7** (50 mg), **8** (5 mg), and **9** (9 mg).

#### Paristenoside A (**1**)

White amorphous powder; $\left[ \alpha \right]_{\text{D}}^{23}$ −76.8 (*c* = 0.05, MeOH); IR (KBr) *v*_max_: 3426, 1631, 1383, 1051 cm^−1^; ^1^H (600 MHz, C_5_D_5_N) and ^13^C NMR (150 MHz, C_5_D_5_N) data, see Tables 1 and 2; ESIMS *m*/*z* 1103 [M + Na]^+^; HRESIMS *m*/*z* 1103.4881 [M + Na]^+^ (cacld for C_50_H_80_O_25_Na, 1103.4881).

#### Paristenoside B (**2**)

White amorphous powder; $\left[ \alpha \right]_{\text{D}}^{23}$ −55.8 (*c* = 0.09, MeOH); IR (KBr) *v*_max_: 3424, 1635, 1382, 1045 cm^−1^; ^1^H (600 MHz, C_5_D_5_N) and ^13^C NMR (150 MHz, C_5_D_5_N) data, see Tables 1 and 2; ESIMS *m*/*z* 1263 [M + Na]^+^; HRESIMS *m*/*z* 1258.5689 [M + NH_4_]^+^ (cacld for C_56_H_92_O_30_N, 1258.5699).

### Sugar Analysis of **1** and **2**

Compounds **1** and **2** (each 2 mg) were refluxed with 2 M HCl (1,4-dioxane/H_2_O 1:1, 2 mL) on water bath for 2 h. The mixture was concentrated in a vacuum, and the residue was suspended in H_2_O and then extracted with CHCl_3_ (5 mL × 3). The aqueous layer was neutralized with MeOH and then dried to give a mixture of sugars. Each mixture was dissolved in anhydrous pyridine (1 mL) and reacted with l-cysteine methyl ester hydrochloride (1.5 mg) stirred at 60 °C for 1.5 h. The trimethylsilylimidazole (1.0 mL) was subsequently added to the reaction mixtures, and they were kept at 60 °C for 30 min. The mixture (4 μL) was finally analyzed by an HP 5890 gas chromatograph with a 30QC2/AC-5 quartz capillary column (30 mm × 0.32 mm × 0.25 *μ*m), respectively, under the following conditions: H_2_ flame ionization detector; carrier gas: N_2_ (1 mL/min); column temperature program: 180–280 °C with the rate of 3 °C/min; injector temperature: 250 °C; split ratio 1:50. Peaks of the hydrolysate were detected by comparison with retention times of authentic samples of d-glucose, d-galactose, d-xylose, and l-rhamnose after the aforementioned treatment in pyridine. The absolute configurations of the sugar moieties were identified as d-glucose (*t*_R_ = 19.0 min), d-galactose (*t*_R_ = 23.0 min), d-xylose (*t*_R_ = 18.3 min), and l-rhamnose (*t*_R_ = 15.4 min), respectively.

### Cytotoxic Assay

Cytotoxic evaluations were performed for two human cancer cell lines (HEK293 and HepG2) using the MTT method described in the literature elsewhere [10]. (−)-OddC was used as the positive control and showed IC_50_ values for the two cancer cell lines with IC_50_ values of 0.30 and 0.17 µM, respectively. The experiments were conducted for three independent replicates, and IC_50_ > 20 μM was considered to be inactive.

## Electronic supplementary material

Below is the link to the electronic supplementary material.

Supplementary material 1 (DOC 1639 kb)
