# Supplementary material for: Two New Highly Oxygenated Spirostanol Saponins from Paris polyphylla var. stenophylla
Source: Nat Prod Bioprospect. 2016 Jun 2;6(4):205–10. doi: 10.1007/s13659-016-0102-x (PMC4940253; doi:10.1007/s13659-016-0102-x)
Supplement: Supplementary file 1 — Supplementary material 1 (DOC 1639 kb) [file 13659_2016_102_MOESM1_ESM.doc]

**Supporting Information for**

**Two New Highly Oxygenated Spirostanol Saponins from *Paris polyphylla* var. *stenophylla***

Ling-Yu Jin ^a, b, 1^, Ting-Xiang Lu ^b, 1^, Xu-Jie Qin ^b^, Wei Ni ^b^, Huan Yan ^b^, Yu Chen ^b, c^,

Hui Liu ^b, c^, Hong-Ping He ^a^, *, Hai-Yang Liu ^b^, *

^a^ College of Pharmacy and Chemistry, Dali University, Dali 671000, China

^b^ State Key Laboratory of Phytochemistry and Plant Resources in West China, Kunming Institute of Botany, Chinese Academy of Science, Kunming 650201, China

^c^ University of Chinese Academy of Science, Beijing 100049, China

*Corresponding author. Tel/Fax: +86-871-65223246.

E-mail address: [95431111@qq.com](mailto:95431111@qq.com). (H.-P. He); [haiyangliu@mail.kib.ac.cn](mailto:haiyangliu@mail.kib.ac.cn). (H.-Y. Liu)

^1^ These authors contributed equally to this work.

.

**Table of Contents**

***Figure S1.*** ^1^H NMR spectrum of paristenoside A (**1**) recorded at 600 MHz in C_5_D_5_N.

***Figure S2.*** ^13^C NMR spectrum of paristenoside A (**1**) recorded at 150 MHz in C_5_D_5_N.

***Figure S3.*** HSQC spectrum of paristenoside A (**1**) recorded in C_5_D_5_N.

***Figure S4.*** HMBC spectrum of paristenoside A (**1**) recorded in C_5_D_5_N.

***Figure S5.*** ^1^H-^1^H COSY spectrum of paristenoside A (**1**) recorded in C_5_D_5_N.

***Figure S6.*** ROESY spectrum of paristenosides A (**1**) recorded in C_5_D_5_N.

***Figure S7.*** HR-ESI-MS spectrum of paristenoside A (**1**).

***Figure S8.*** ^1^H NMR spectrum of paristenoside B (**2**) recorded at 600 MHz in C_5_D_5_N.

***Figure S9.*** ^13^C NMR spectrum of paristenoside B (**2**) recorded at 150 MHz in C_5_D_5_N.

.***Figure S10.*** HSQC spectrum of paristenoside B (**2**) recorded in C_5_D_5_N.

***Figure S11.*** HMBC spectrum of paristenoside B (**2**) recorded in C_5_D_5_N.

***Figure S12.*** ^1^H-^1^H COSY spectrum of paristenoside B (**2**) recorded in C_5_D_5_N.

***Figure S13.*** ROESY spectrum of paristenoside B (**2**) recorded in C_5_D_5_N.

***Figure S14.*** HR-ESI-MS spectrum of paristenoside B (**2**).

***Figure S15.*** Chemical structures of compounds **3**‒**9**.

**
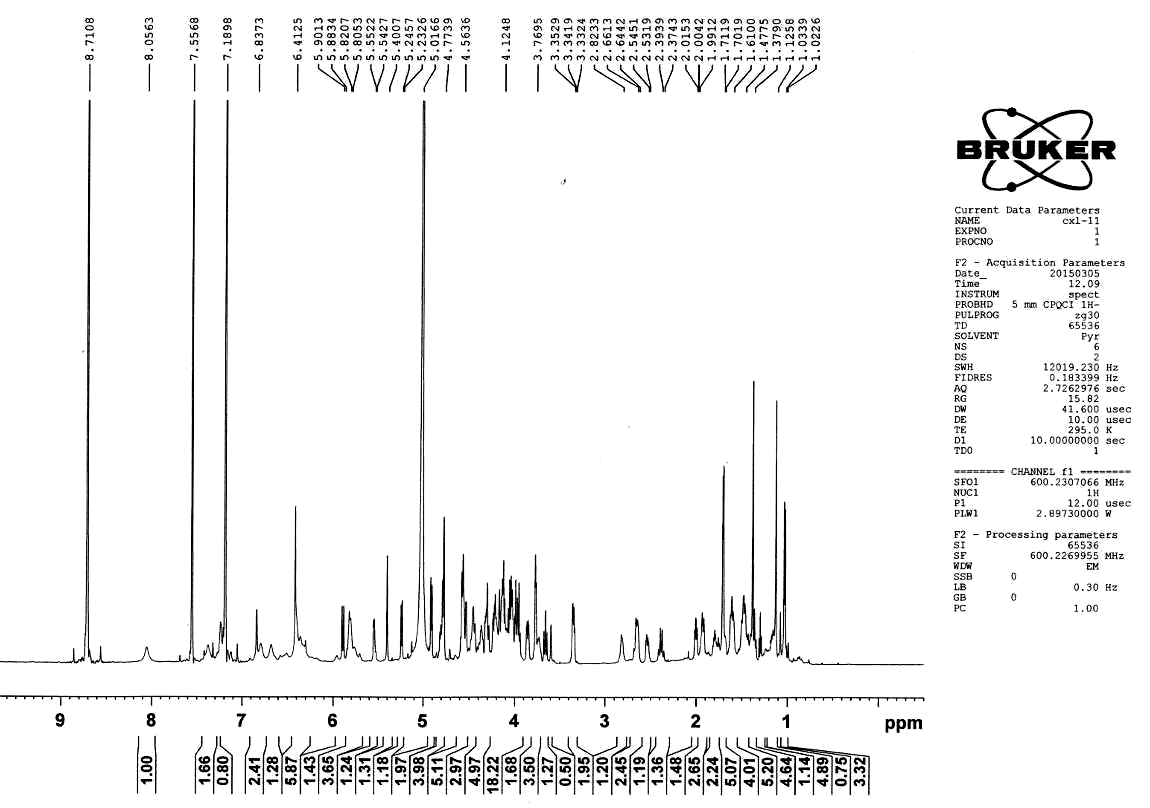
**

***Figure S1.*** ^1^H NMR spectrum of paristenoside A (**1**) recorded at 600 MHz in C_5_D_5_N

**
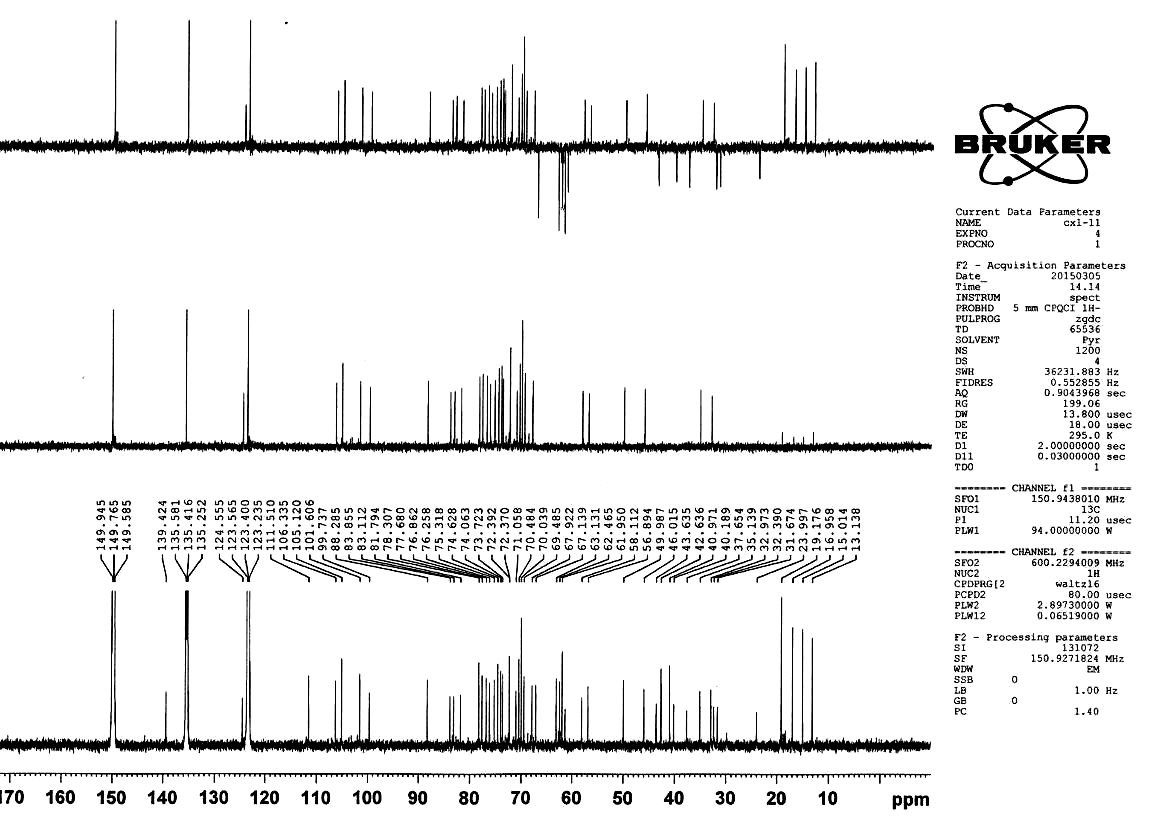
**

***Figure S2.*** ^13^C NMR spectrum of paristenoside A (**1**) recorded at 150 MHz in C_5_D_5_N


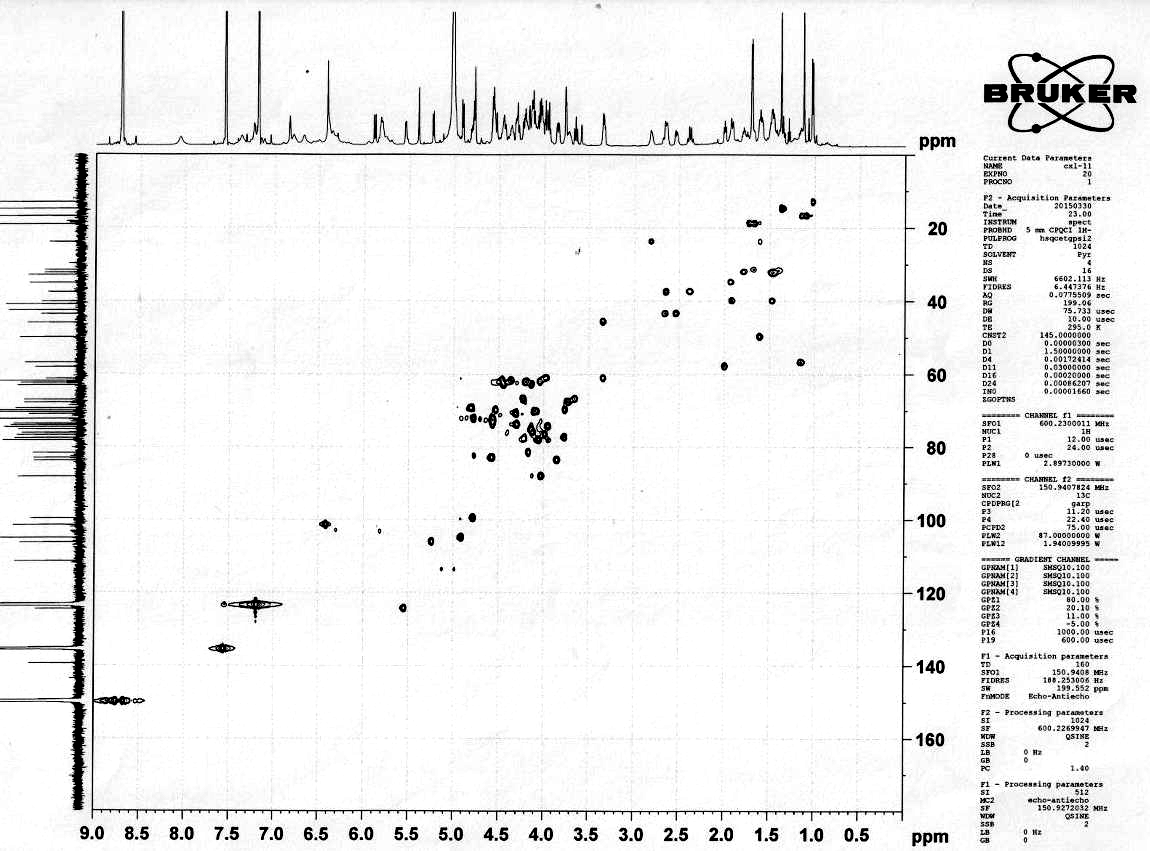


***Figure S3.*** HSQC spectrum of paristenoside A (**1**) recorded in C_5_D_5_N


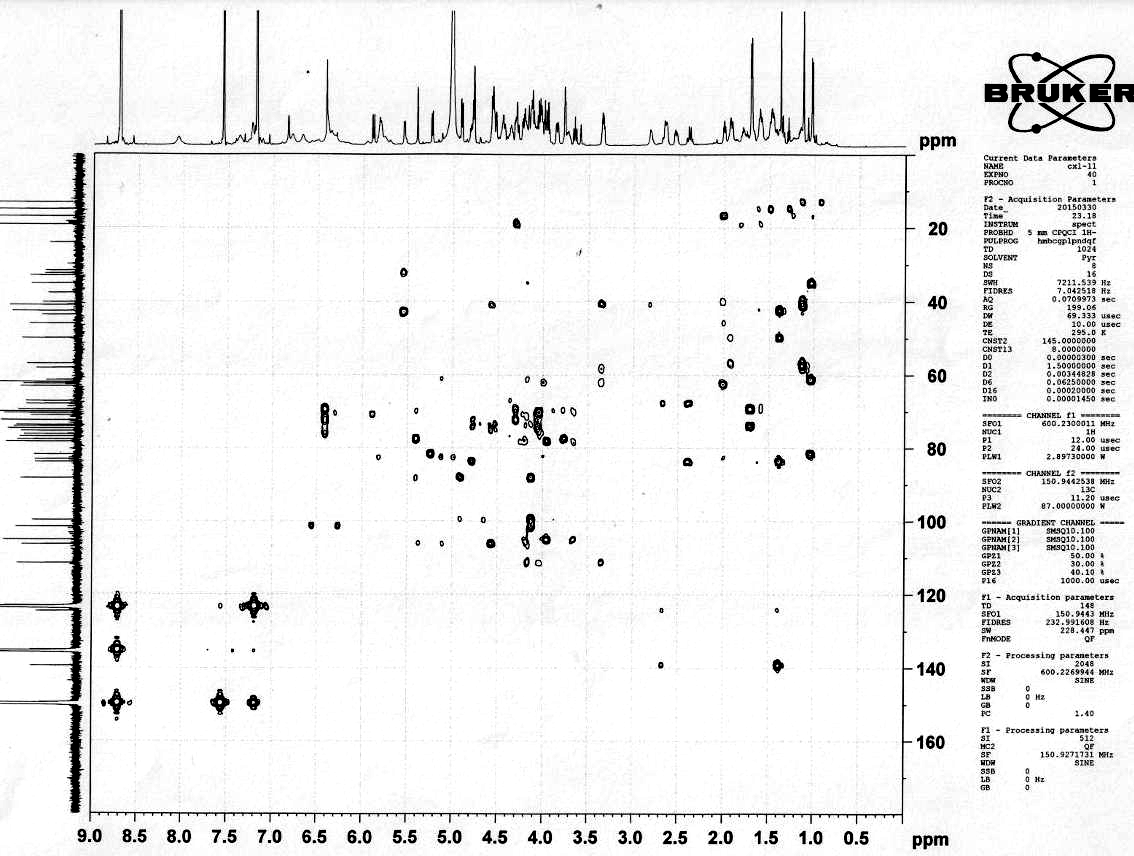


***Figure S4.*** HMBC spectrum of paristenoside A (**1**) recorded in C_5_D_5_N

***
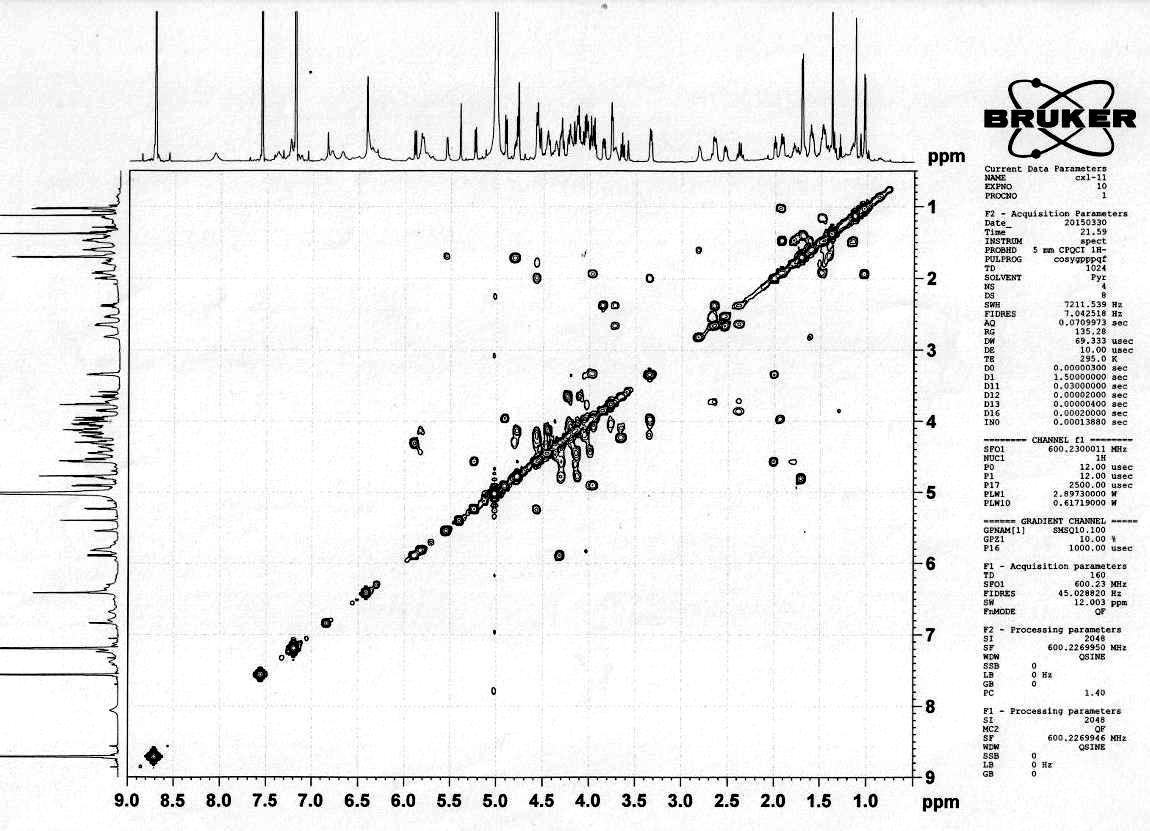
***

***Figure S5.*** ^1^H-^1^H COSY spectrum of paristenoside A (**1**) recorded in C_5_D_5_N

***
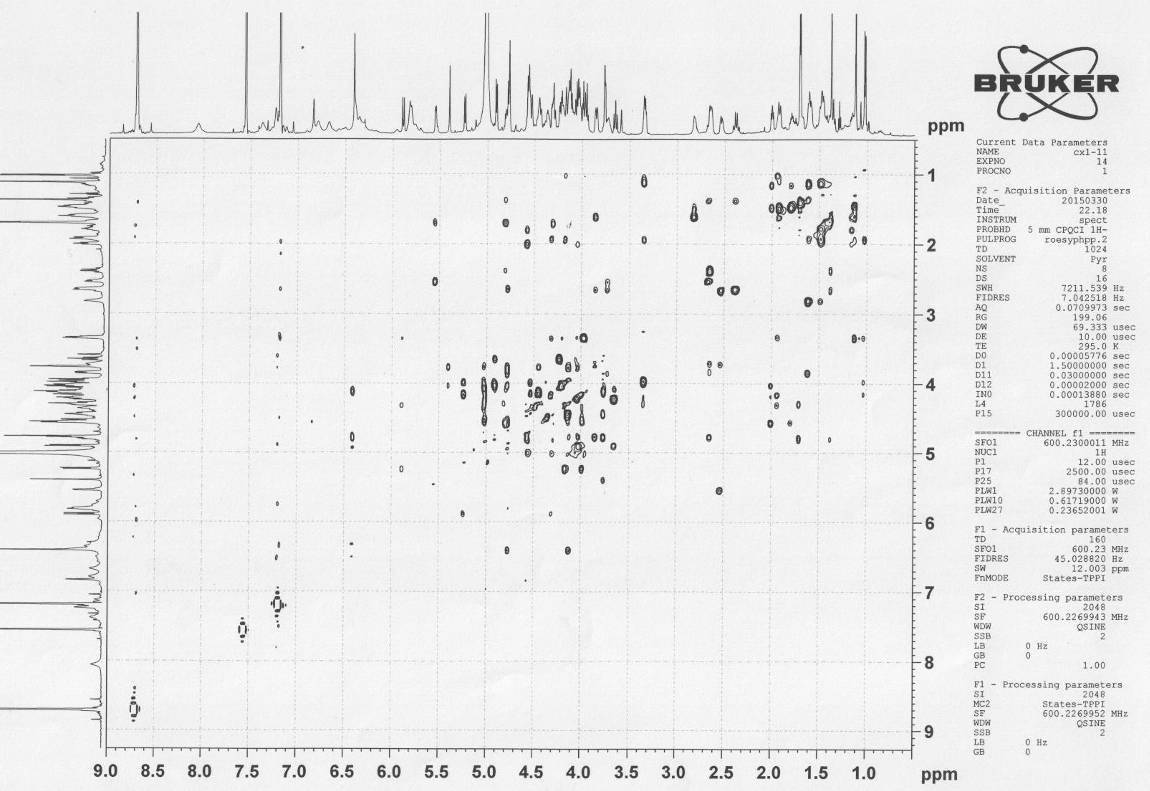
***

***Figure S6.*** ROESY spectrum of paristenosides A (**1**) recorded in C_5_D_5_N

***
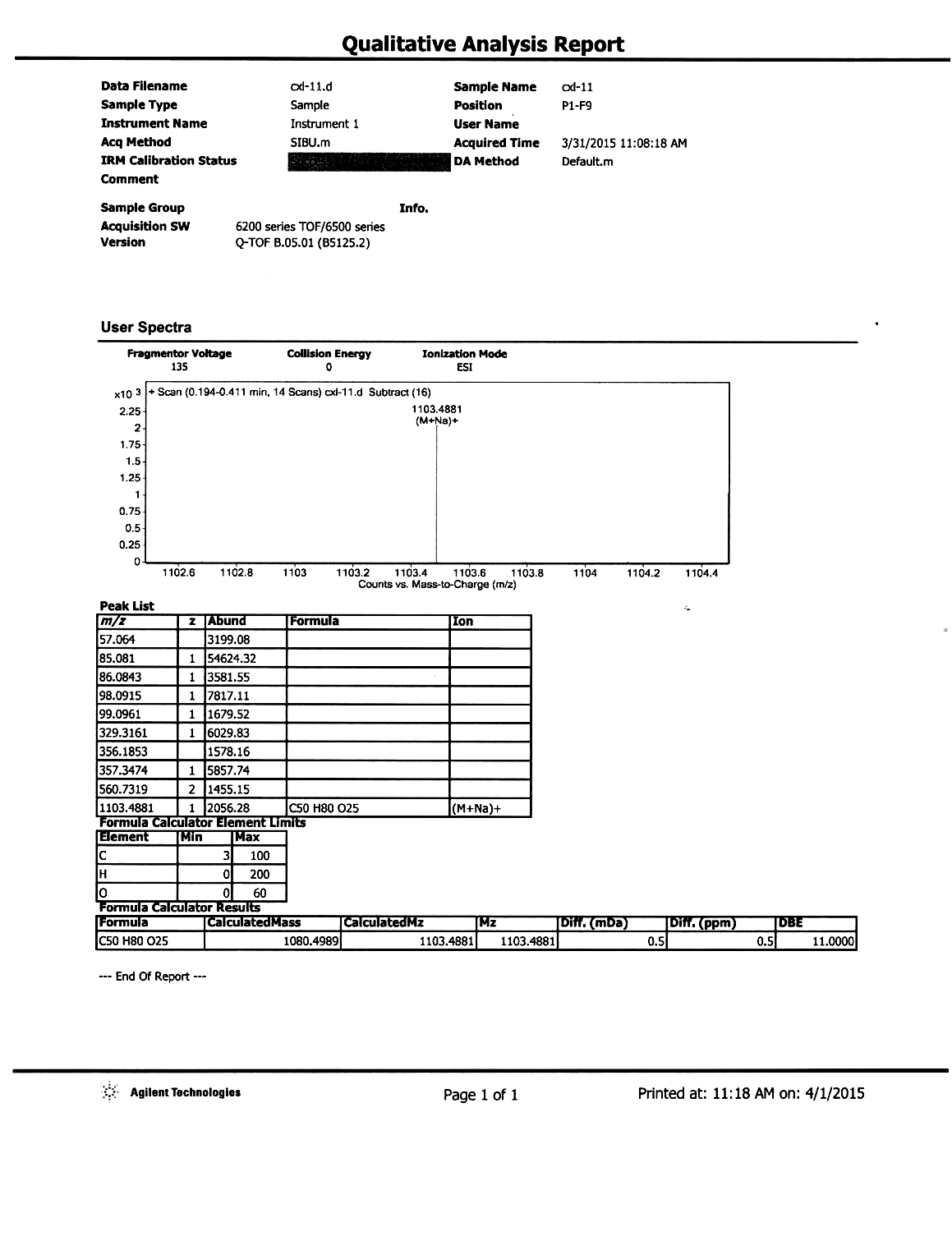
***

***Figure S7.*** HRESIMS spectrum of paristenoside A (**1**)


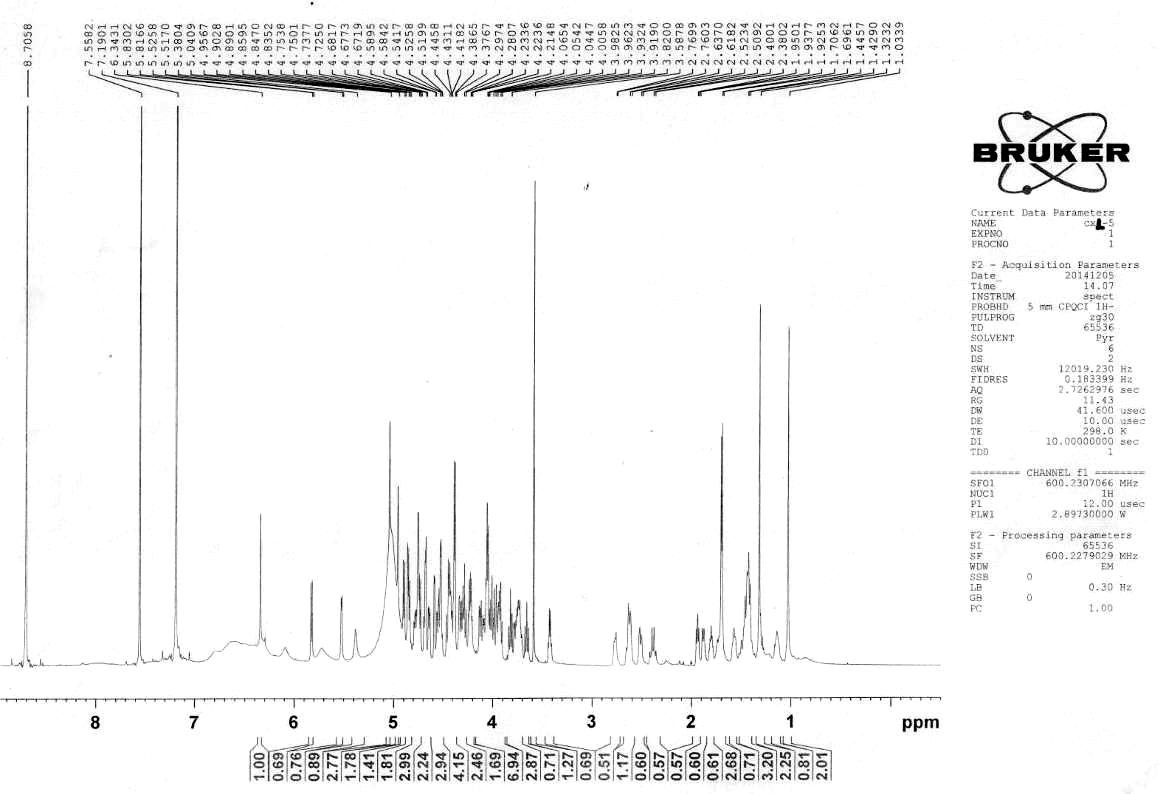


***Figure S8.*** ^1^H NMR spectrum of paristenoside B (**2**) recorded at 600 MHz in C_5_D_5_N


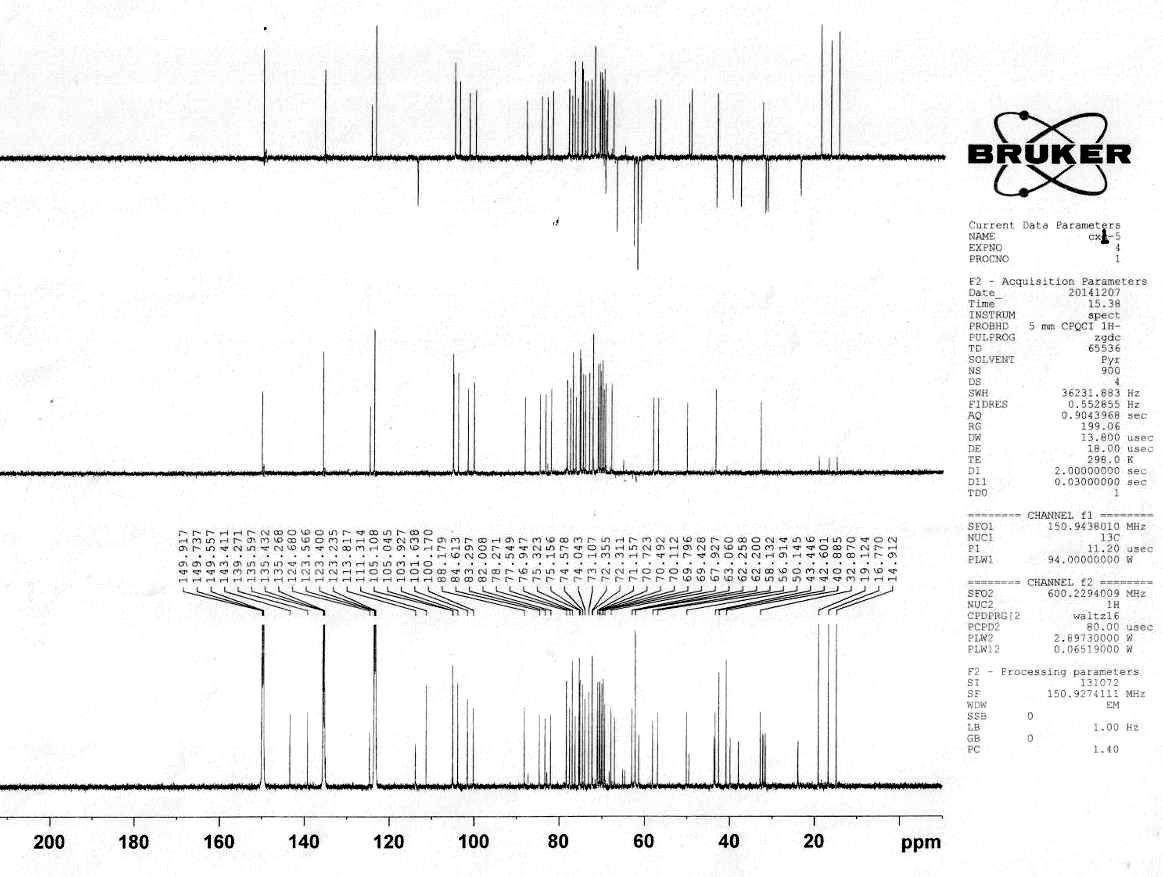


***Figure S9.*** ^13^C NMR spectrum of paristenoside B (**2**) recorded at 150 MHz in C_5_D_5_N


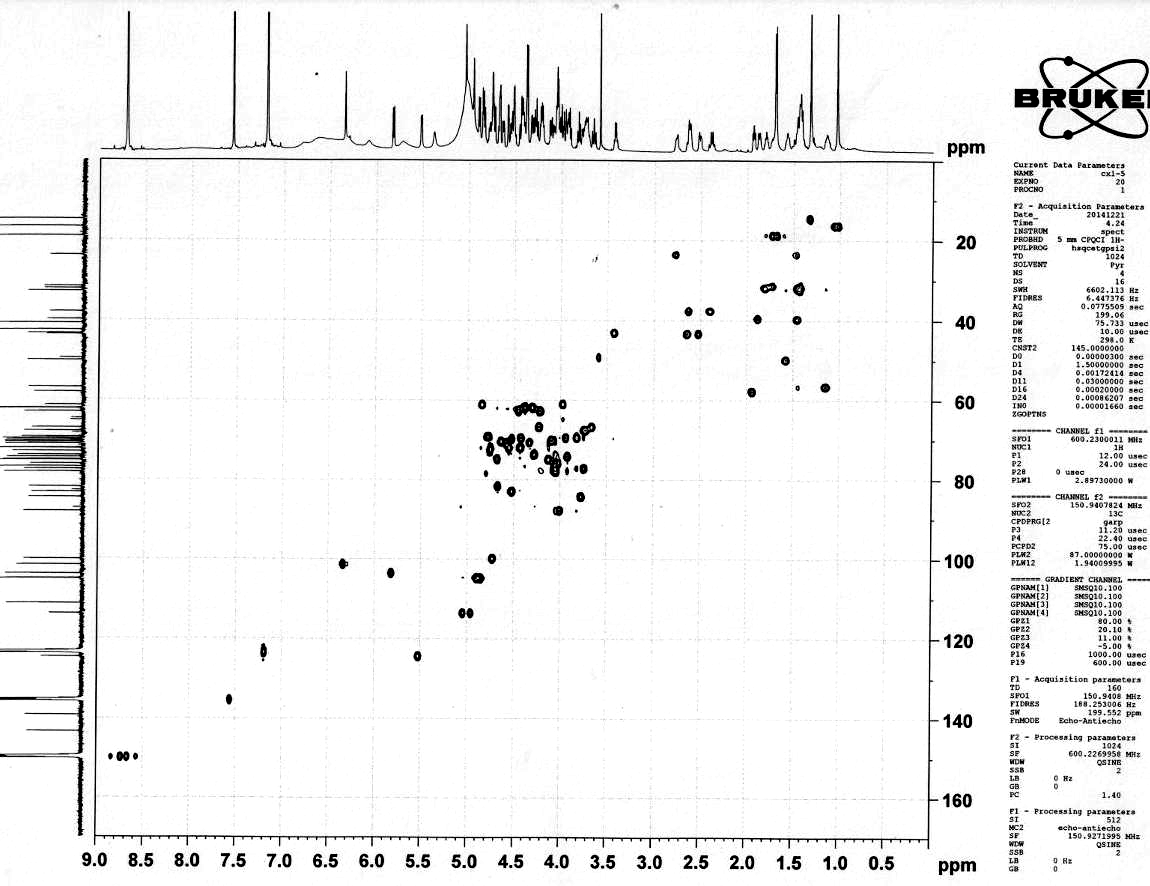


***Figure S10.*** HSQC spectrum of paristenoside B (**2**) recorded in C_5_D_5_N


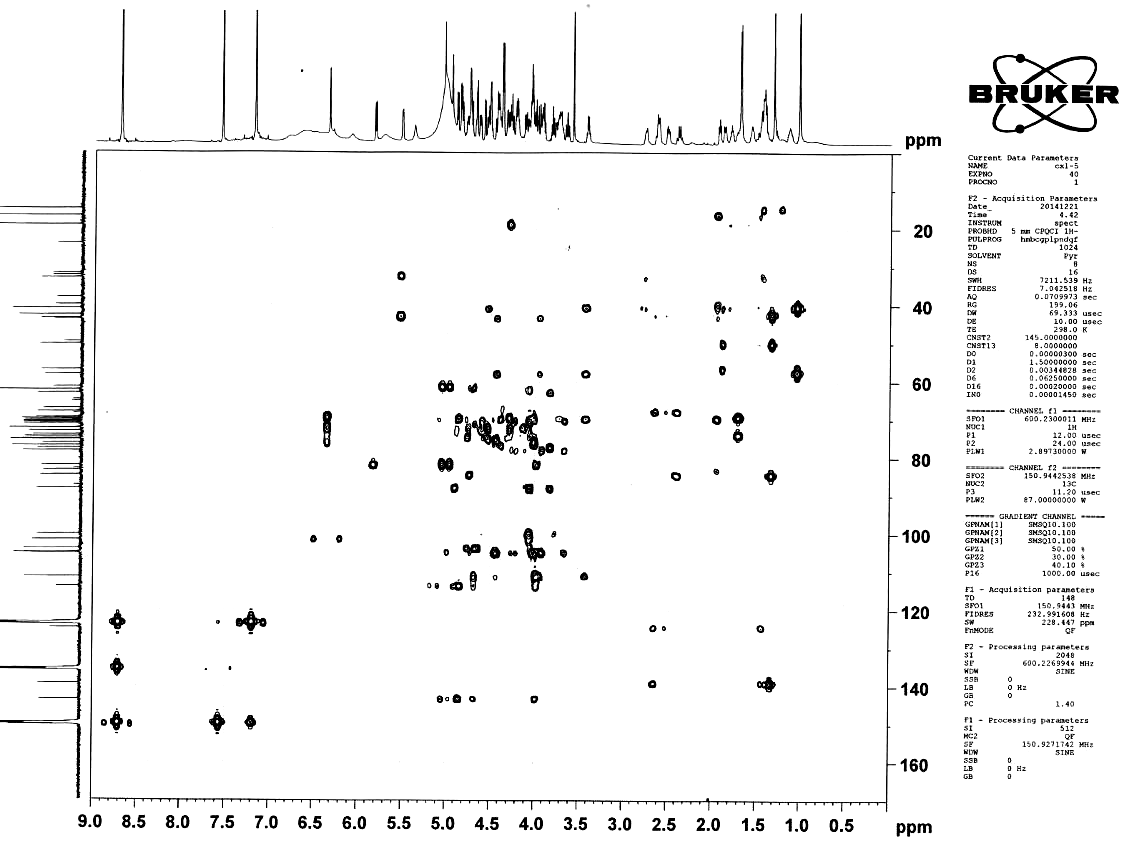


***Figure S11.*** HMBC spectrum of paristenoside B (**2**) recorded in C_5_D_5_N

***
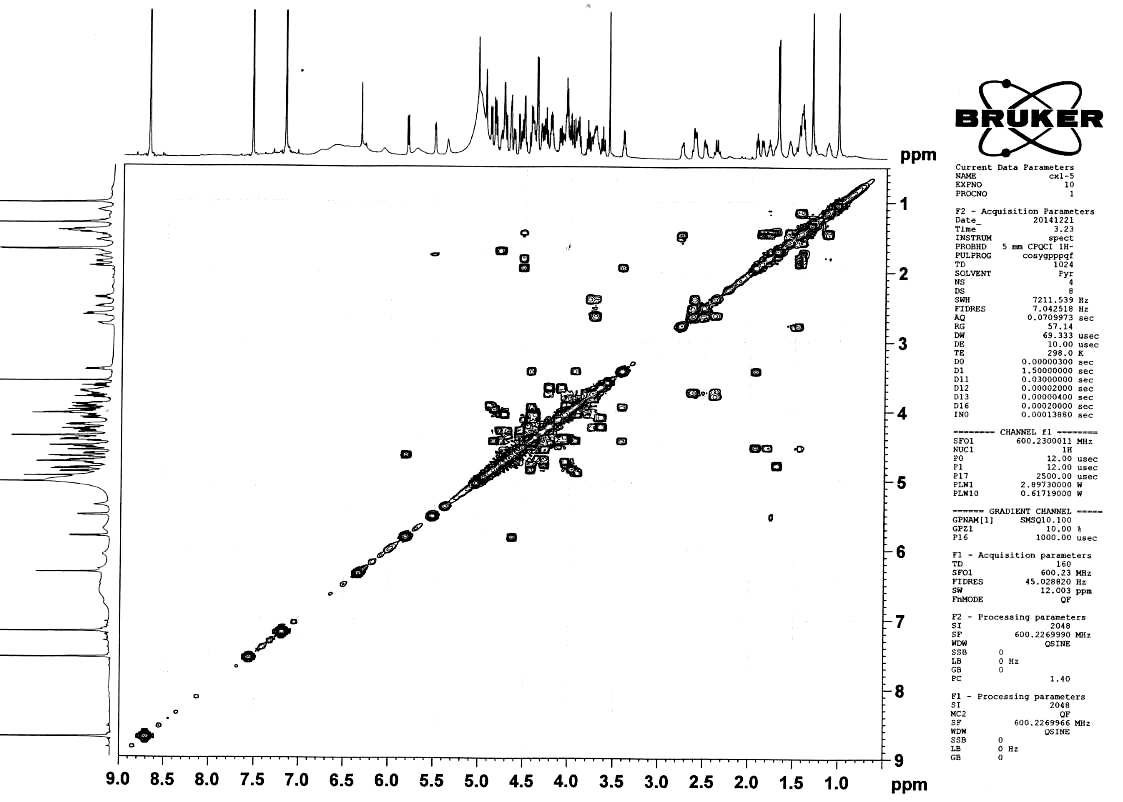
***

***Figure S12.*** ^1^H-^1^H COSY spectrum of paristenoside B (**2**) recorded in C_5_D_5_N


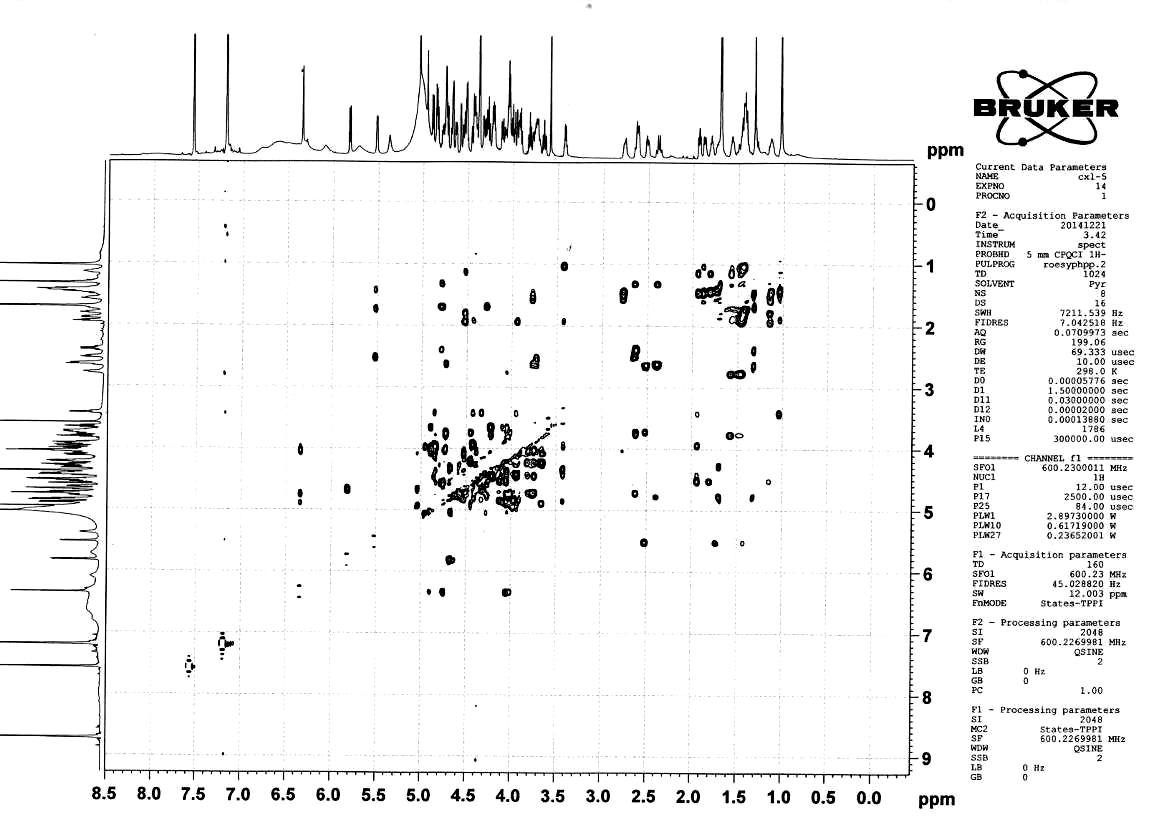


***Figure S13.*** ROESY spectrum of paristenoside B (**2**) recorded in C_5_D_5_N

***
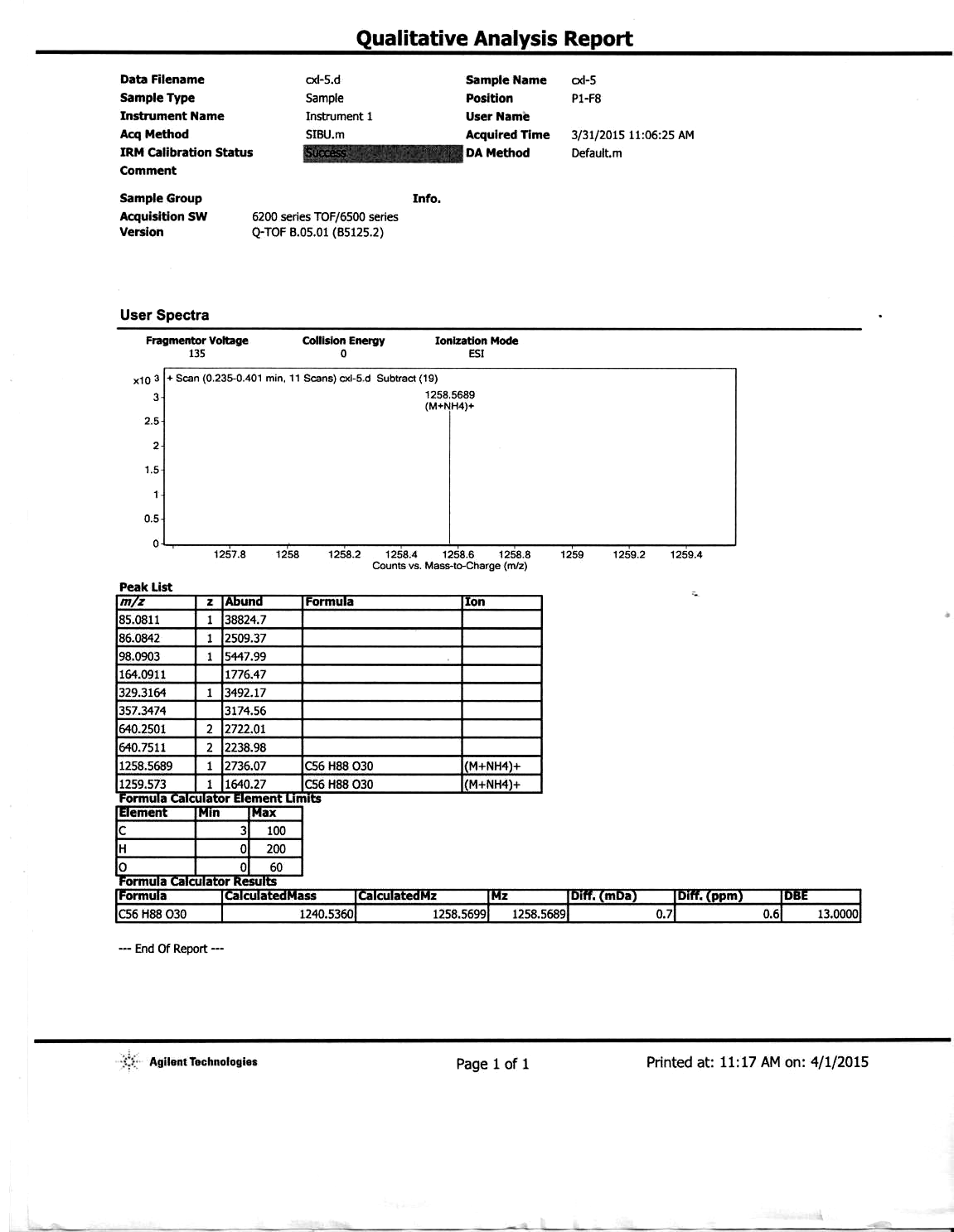
***

***Figure S14.*** HRESIMS spectrum of paristenoside B (**2**)

***Figure S15.*** Chemical structures of compounds **3**‒**9**.
